# LOW BACK PAIN ESTIMATES IN PROFESSIONAL SOCCER: A SYSTEMATIC REVIEW AND META-ANALYSIS

**DOI:** 10.1590/1413-785220233105e266012

**Published:** 2023-12-18

**Authors:** JULIANO BERGAMASCHINE MATA DIZ, MARIA THERESA PEREIRA DUTRA, ISABELLA CHEREMETTA FEIJÓ, ANA LUÍZA MORAIS SOGNO, FERNANDA REZENDE SILVA, GIOVANNA DE FARIA CARNEVALE, BRUNO DE SOUZA MOREIRA, CARLOS FERNANDO MOREIRA SILVA

**Affiliations:** 1 Faculdade de Medicina de Barbacena, Barbacena, MG, Brazil; 2 Universidade Federal de Minas Gerais, Belo Horizonte, MG, Brazil

**Keywords:** Low Back Pain, Epidemiology, Prevalence, Sports, Soccer, Professional Athletes, Lombalgia, Epidemiologia, Prevalência, Esportes, Futebol, Atletas Profissionais

## Abstract

**Objective:**

To evaluate the epidemiological and clinical characteristics of low back pain (LBP) in adult professional soccer players.

**Methods:**

Systematic review and meta-analysis.

**Results:**

The review included 44 studies. The pooled prevalence of LBP during ≤ 1 season was 1% (95%CI = 0-4%) in men. The pooled point prevalence of LBP was 25% (95%CI = 16-36%) in men and 28% (95%CI = 20-37%) in women. The pooled past-year prevalence of LBP was 34% (95%CI = 24-44%) in men. The pooled lifetime prevalence of LBP was 32% (95%CI = 25-39%) in men and 50% (95%CI = 32-69%) in women. The pooled frequency of LBP/total number of injuries was 2% (95%CI = 1-3%) in men and 4% (95%CI = 2-5%) in women. The pooled incidence rate of LBP/1,000 player-hours of exposure was 0.30 (95%CI = 0.17- 0.53) in men and 0.32 (95%CI = 0.06 -1.87) in women. The recurrence of LBP ranged from 3% to 63% in men. The intensity of LBP ranged from 1.68 (2.39) to 4.87 (2.14) points on a 0-10 scale (minimum = 0 and maximum = 8 points). The severity of LBP (days absent from professional activities due to pain) ranged from 2 (0) to 10 (19) days (minimum = 1 and maximum = 28 days).

**Conclusion:**

Adult elite soccer players have a substantial prevalence of LBP. The frequency and incidence of LBP (compared with other conditions and sports) seems to be low. Estimates of the recurrence, intensity, and severity of LBP are uncertain. ***Level of Evidence II, Systematic Review of Level II Studies.***

## RESUMO

**Objetivo:**

Investigar as características epidemiológicas e clínicas da lombalgia em jogadores profissionais de futebol.

**Métodos:**

Revisão sistemática e metanálise.

**Resultados:**

A revisão incluiu 44 estudos. A prevalência combinada de lombalgia em até uma temporada foi de 1% (IC95% = 0-4%) em homens. A prevalência pontual combinada de lombalgia foi de 25% (IC95% = 16-36%) em homens e 28% (IC95% = 20-37%) em mulheres. A prevalência combinada de lombalgia no último ano foi de 34% (IC95% = 24-44%) em homens. A prevalência combinada de lombalgia ao longo da vida foi de 32%(IC95% = 25-39%) em homens e 50% (IC95% = 32-69%) em mulheres. A frequência combinada de lombalgia/número total de lesões foi de 2% (IC95% = 1-3%) em homens e 4% (IC95% = 2-5%) em mulheres. A taxa de incidência combinada de lombalgia/1.000 jogador-horas de exposição foi de 0,30 (IC95% = 0,17-0,53) em homens e 0,32 (IC95% = 0,06-1,87) em mulheres. A recorrência de lombalgia variou entre 3-63% em homens. A intensidade da lombalgia variou entre 1,68 (2,39)-4,87 (2,14) pontos em uma escala de 0-10 (mínimo = 0; máximo = 8 pontos). A gravidade da lombalgia (ausência das atividades profissionais devido à dor) variou entre 2 (0)-10 (19) dias (mínimo = 1; máximo = 28 dias).

**Conclusão:**

Jogadores de futebol profissional apresentam alta prevalência de lombalgia substancial. A frequência e a incidência da lombalgia parecem ser baixas comparadas a outros esportes e condições. As estimativas de recorrência, intensidade e gravidade da lombalgia são incertas. **Nível de Evidência II, Revisão Sistemática de Estudos de Nível II.**

## INTRODUCTION

Low back pain (LBP) is a common complaint in the general population and represents one of the main causes of seeking medical care worldwide.^1^ It is associated with high rates of physical disability and work absenteeism, and therefore has a huge negative socio-economic effect on patients and health systems, both public and private. ^(^ ^2^ This condition has a multifactorial etiology, and a wide range of biopsychosocial factors may contribute to the onset and improvement or worsening of patients’ signs/symptoms. ^(^ ^3^ Professional athletes, regardless of their sport, often experience LBP, since the level of physical and psychological demand in training and competitions is significantly higher than in non-athletes. ^(^ ^4^ Previous systematic reviews on the epidemiology of LBP in sports showed point prevalence estimates ranging from 10% to 67% and 12-month prevalence estimates ranging from 17% to 94%.^4^ ^),(^ ^5^ Thus, the clinical approach to athletes with back complaints involves permanent care that goes beyond relieving symptoms and restoring functionality. Screening for potential risk factors that may predispose to back pain during sports practice is necessary in order to suppress or attenuate causal mechanisms and prevent recurrences. ^(^ ^6^ Moreover, when professional athletes have a musculoskeletal problem, they need to recover as quickly as possible, fully restoring their physical and functional capabilities to train/compete at the highest levels of performance. ^(^ ^7^ However, besides the need for athletes to fully recover in time for their professional commitments, institutions (e.g., clubs and federations) impose burdens arising from the absence of athletes in their activities, whether financial costs or burdens directly related to the inability of athletes to perform in commitments on the official calendar. ^(^ ^8^

Soccer, one of the most popular sports in the world, exposes its players to high mechanical stress, such as repetitive movements, excessive loads, and high-energy trauma. This can easily affect the musculoskeletal system, especially the lumbar spine, which is one of the body regions most susceptible to dysfunction due to traumatic, overuse, and/or degenerative mechanisms. ^(^ ^6^ ^),(^ ^9^ Especially considering professional soccer and the level of performance it has reached in the contemporary sports world, studying LBP in this context can evidence its negative repercussions for athletes and institutions and provide important support for pain prevention and management strategies. Thus, this study aimed to evaluate the epidemiological (prevalence and incidence) and clinical (recurrence and severity) characteristics of LBP in professional soccer players.

## METHODS

### Study design and guidelines

This is a systematic review and meta-analysis. Its methods were based on recommendations of the JBI Manual for Evidence Synthesis, ^(^ ^10^ the Meta-analysis of Observational Studies in Epidemiology (MOOSE) group, ^(^ ^11^ and the Cochrane Handbook for Systematic Reviews of Interventions. ^(^ ^12^ The review followed the Preferred Reporting Items for Systematic Reviews and Meta-Analyses (PRISMA) checklist^13^ and the Prisma in Exercise, Rehabilitation, Sport Medicine and Sports Science (PERSiST) guidance. ^(^ ^14^ PROSPERO No. CRD42021271942.

### Search strategy and inclusion criteria

Searches for original studies were conducted in the Embase, LILACS, PubMed/MEDLINE, SciELO, Scopus, SPORTDiscus, and Web of Science databases, without date or language restrictions. A manual search was also performed in Google Scholar, specialized scientific journals, and reference lists of previous studies. Moreover, professionals/researchers in the field were consulted to identify additional relevant records. Search strategies were elaborated using combinations of descriptors/terms for each database, using English words such as “epidemiology,” “prevalence,” “incidence,” “backache,” “spine,” “injury,” “sport,” “football,” “soccer,” “athlete,” “professional,” and “elite.” Supplementary Table 1 presents detailed search strategies.

Studies with data on LBP in adult professional soccer players of both sexes, regardless of academic type (e.g., conference abstract, dissertation/thesis, or article) and design (e.g., observational or experimental), were the inclusion criteria. Anatomically, LBP is any pain and/or discomfort in the region between the costal margin and the inferior gluteal folds, with or without radiation to the lower limbs, regardless of the cause (specific or non-specific) and evolution (acute or chronic). ^(^ ^15^ ^),(^ ^16^ The sport assessed was the traditional field soccer^17^ in professional contexts involving seasons, training, and/ or competitions (e.g., matches, tournaments, championships, leagues, and cups). No minimum sample size was considered as an inclusion criterion in order to increase the number of eligible studies. Studies with other types of soccer (e.g., indoor, beach, and Paralympic), different age groups (e.g., children and young people), and non-professional levels (e.g., amateur athletes) were excluded.

### Study selection and data extraction

Two reviewers independently screened the titles and abstracts of the original studies obtained from the searches. The full texts of potential studies were accessed and assessed for eligibility. The studies that met the inclusion criteria were included in the review. Data were extracted by two independent reviewers to avoid the omission of relevant data. Disagreements were resolved by consensus. ^(^ ^10^ The following information was extracted: study (author and date); location (country); design (cross-sectional or longitudinal) sample (size and sex and age of participants); assessment time [during a season (≤ 12 months), for longer than a season (> 12 months), or during a given time (e.g., point) and/ or period (e.g., past year)]; exposure (total hours of exposure in training and/or matches); injury (total number of soccer-related injuries); and outcome (prevalence and/or incidence). The authors of original studies were contacted via email to clarify unclear/ missing information and/or provide additional data.

### Risk of bias assessment

Two reviewers independently assessed the risk of bias of each included study, using a tool developed by Loney and Stratford^18^ and Loney et al., ^(^ ^19^ which has eight items that address methodological issues of prevalence/incidence studies. This tool was chosen because it best applies to the scope of this review (considering its condition, context, and population). ^(^ ^10^ Items 1 and 2 refer to the study design, the description of the setting, and the characteristics of participants. Items 3 and 4 refer to sample selection and size. Items 5 to 7 refer to diagnostic methods, data collection, and statistical analysis, and item 8 refers to the response rate and the follow-up period. ^(^ ^18^

For evaluation purposes, in item 1, a cross-sectional design was considered adequate for prevalence studies and a longitudinal design (prospective or retrospective) for incidence studies. ^(^ ^18^ ^),(^ ^19^ In item 2, the clear presentation of the origin, affiliation, and characteristics of participants was considered adequate. ^(^ ^18^ ^),(^ ^19^ In item 3, a sample selection by convenience from professional soccer settings, such as clubs, national teams, and/or competitions, was considered acceptable. ^(^ ^20^ In item 4, a sample size of ≥ 25 participants was considered adequate, as this is the average number of players at a professional soccer club during a season and/or competition. ^(^ ^21^ ^),(^ ^22^ In items 5 and 6, the identification of LBP cases/events using standardized records with sufficient information on the assessment, exposure, and outcome, according to the definitions of injury resulting from soccer suggested by Fuller et al., ^(^ ^20^ Hägglund et al., ^(^ ^23^ and Timpka et al. ^(^ ^24^ (e.g., inability to play/train; need for medical care; detectable tissue damage; or self-reported complaint resulting from injury) was considered adequate. In item 7, an explicit reporting of prevalence/ incidence results with confidence intervals (CI) was considered adequate. ^(^ ^18^ ^),(^ ^19^ In item 8, a response rate ≥ 70% was considered acceptable, ^(^ ^18^ ^),(^ ^19^ while for incidence studies, the acceptable follow-up period should cover at least one full official tournament, ^(^ ^20^ with a sample loss < 20%.^25^

For each item in the assessment tool, the answer was “yes,” “unclear,” or “no,” depending on whether the information in the included studies was sufficiently clear, obscure, or absent, respectively. The answer “yes” was classified as “low risk of bias;” “unclear” as “unknown risk of bias;” and “no” as “high risk of bias.” Disagreements were resolved by a third reviewer. ^(^ ^10^ The authors of original studies were contacted via email if additional information was required. The frequency of answers for each item was estimated and presented in a bar chart. A total average of “low risk of bias” answers was provided without, however, using it as a selection or judgment criterion.

### Data analysis and evidence synthesis

The data from each included study were initially described using descriptive statistics. Study-level prevalence estimates were obtained using the formula: ^(^ ^26^

$$\mathrm{Prevalence}=\frac{\mathrm{number}\;\mathrm{of}\;\mathrm{positive}\;\mathrm{LBP}\;\mathrm{cases}}{\mathrm{total}\;\mathrm{number}\;\mathrm{of}\;\mathrm{players}\;\mathrm{in}\;\mathrm{the}\;\mathrm{study}}\times 100$$

Study-level injury frequencies were obtained by the formula: ^(^ ^26^

$$\mathrm{Frequency}=\frac{\mathrm{number}\;\mathrm{of}\;\mathrm{positive}\;\mathrm{LBP}\;\mathrm{cases}}{\mathrm{total}\;\mathrm{number}\;\mathrm{of}\;\mathrm{injuries}\;\mathrm{in}\;\mathrm{the}\;\mathrm{study}}\times 100$$

Study-level incidence rates were obtained by the formula: ^(^ ^20^ ^),(^ ^26^

$$\mathrm{Incidence}=\frac{\mathrm{number}\;\mathrm{of}\;\mathrm{positive}\;\mathrm{LBP}\;\mathrm{cases}}{\mathrm{total}\;\mathrm{exposure}\;(\mathrm{in}\;\mathrm{hours})\;\mathrm{in}\;\mathrm{the}\;\mathrm{study}}\times 1,000\;\mathrm{hours}\;\mathrm{of}\;\mathrm{exposure}$$

For prevalence and injury frequency estimates, a 95%CI was estimated using the Wilson method for n ≤ 40 and the Agresti-Coull method for n > 40, while for incidence rates, a 95%CI was estimated using the Clopper-Pearson exact method. ^(^ ^27^ All descriptive analyses were performed using the EpiTools epidemiological calculator (Ausvet, 2018; https://epitools.ausvet.com.Au/ciproportion).

The meta-analysis of injury prevalence and frequency was conducted by pooling the proportions obtained in the included studies, using the inverse variance heterogeneity (Ivhet) model, which estimates the variance of the pooled effect by a quasi-likelihood framework. ^(^ ^28^ ^),(^ ^29^ This model has shown better performance in reducing the observed variance and improving the accuracy of estimates compared with the traditional DerSimonian-Laird random effects model, ^(^ ^30^ especially when the number of pooled studies is small (e.g., k < 10) and the heterogeneity is substantially high (e.g., I^2^ > 50%).^28^ ^),(^ ^29^ Moreover, the proportions were normalized using the Freeman-Tukey double arcsine transformation in order to stabilize the variance within/between studies when estimating study weights. ^(^ ^31^ This approach improves variance estimation in analyses that include studies with small sample sizes and proportions close to 0.0 or 1.0. ^(^ ^10^ ^),(^ ^31^

The meta-analysis of incidence was conducted by pooling the rates and their respective standard errors obtained in the included studies, using the DerSimonian-Laird random effects model. ^(^ ^30^ Rates were expressed per 1,000 player-hours of exposure, according to the formula: ^(^ ^32^

$$\mathrm{Incidence}=\frac{\mathrm{number}\;\mathrm{of}\;\mathrm{positive}\;\mathrm{LBP}\;\mathrm{events}}{\mathrm{number}\;\mathrm{of}\;\mathrm{matches}\;x\;\mathrm{number}\;\mathrm{of}\;\mathrm{players}\;x\;\mathrm{match}\;\mathrm{length}}\times 1,000\;\mathrm{hours}\;\mathrm{of}\;\mathrm{exposure}$$

Data on exposure in training and/or matches were obtained from the included studies. When an incidence rate was not provided in the studies that reported injuries during competitions, the number of positive LBP events, the number of matches, the number of exposed players (11 or 22), and match length in hours (90 minutes = 1.5 hours), were used to obtain incidence rates, as in the formula above. ^(^ ^20^ ^),(^ ^32^

Heterogeneity between pooled studies was assessed using Cochran’s Q test. A large Q value with p < 0.10 suggests the presence of significant heterogeneity. Quantification of variability (%) was assessed using the I^2^ statistic, and a value ≥ 75% showed considerable heterogeneity. ^(^ ^12^ Publication bias was assessed for meta-analyses with k ≥ 10 studies using the Doi plot method. ^(^ ^33^ For quantification of asymmetry, the LFK index was used. A value less than or equal to ± 1 represented “absent asymmetry” (absent publication bias), a value between ± 1 and ± 2 represented “minor asymmetry” (present publication bias), and a value greater than ± 2 represented “major asymmetry” (significant publication bias). Moreover, Egger’s test with p < 0.10 was used as an additional inference of significant asymmetry. ^(^ ^33^ All meta-analyses were performed using MetaXL software version 5.3 (EpiGear International Pty Ltd., Sunrise Beach, Queensland, Australia, 2016).

The quality of evidence for prevalence estimates, injury frequencies, and incidence rates was rated by two independent reviewers using the Grading of Recommendations Assessment, Development, and Evaluation (GRADE) system. ^(^ ^34^ The levels of quality of evidence were: high quality (the pooled estimates/rates are very close to the actual estimates/rates, and differences are unlikely); moderate quality (the pooled estimates/rates are close to the actual estimates/rates, but may differ); low quality (the pooled estimates/rates are uncertain and likely to differ from the actual estimates/rates); and very low quality (the pooled estimates/rates are very uncertain and probably very different from the actual estimates/rates).

The overall quality of evidence for each pooled result was initially rated as high and then downgraded by one, two, or three levels (up to very low) if one of the following criteria were present: ≥ 50% of pooled studies were classified as “high risk of bias” in items 4, 5, or 6 of the tool (serious risk of bias); ≥ 50% of pooled studies did not use valid/reliable methods to identify LBP in soccer settings^20^ (serious indirectness); ≥ 50% of pooled studies did not have a sample of 25 participants or more (serious imprecision); the I^2^ of the pooled analysis was ≥ 75% (serious inconsistency); and the analysis of publication bias showed “major asymmetry” and Egger’s test with p < 0.10 (serious publication bias). ^(^ ^34^ For meta-analyses with k < 10 studies, the analysis of publication bias was not conducted and therefore not used as a criterion for rating the quality of evidence. Finally, the clinical features of LBP were described as follows: recurrence rate (%); pain intensity (average points on a 0-10 scale and categorization into three levels: ≤ 3 points = mild; 4-7 points = moderate; 8-10 points = severe); and pain severity (average number of days a player is absent from professional activities due to pain, from the first day absent until full return to training/matches, and categorization into four levels: ≤ 3 days = minimal; 4-7 days = mild; 8-28 days = moderate; > 28 days = severe). ^(^ ^15^ ^),(^ ^20^

## RESULTS

### Study selection process

**Figure 1:**
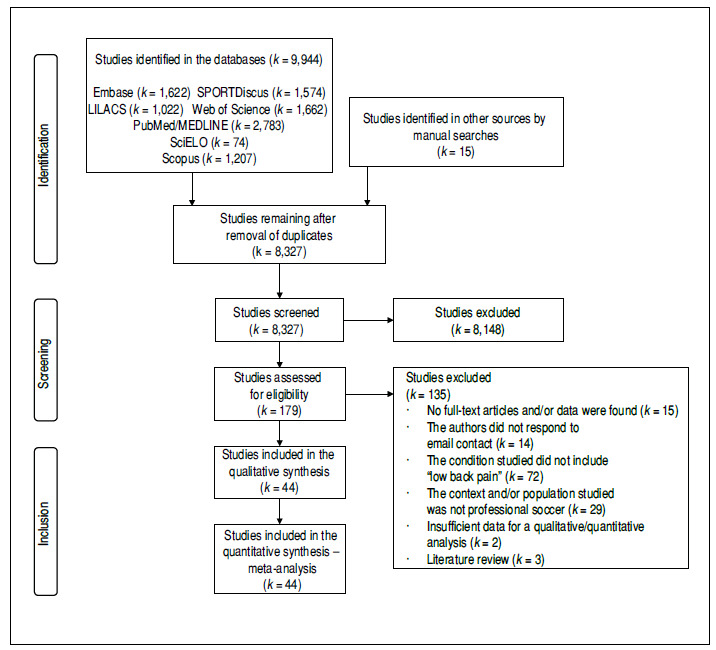
PRISMA flowchart of studies in the review (k = 44).

The searches identified 9,959 studies. We removed 1,632 duplicates and excluded 8,148 based on their titles/abstracts. We read 179 original studies in full and assessed their eligibility. Finally, we excluded 135 for six different reasons and included 44 in this review^22^ ^),(^ ^35^ ^)-(^ ^79^ (Figure 1). The study by van Beijsterveldt et al. ^(^ ^75^ used data from the same sample as the study by Stubbe et al. ^(^ ^71^ The PhD dissertations by Hägglund^52^ and Netto^61^ only provided additional data on their respective original articles. ^(^ ^51^ ^), (^ ^60^

### Study description

The included studies were published from 1991 to 2021 and conducted in Europe (k = 27), ^(^ ^22^ ^),(^ ^35^ ^)-(^ ^40^ ^),(^ ^44^ ^),(^ ^46^ ^)-(^ ^48^ ^),(^ ^50^ ^),(^ ^51^ ^),(^ ^54^ ^)-(^ ^57^ ^),(^ ^64^ ^),(^ ^67^ ^),(^ ^68^ ^),(^ ^71^ ^),(^ ^73^ ^)-(^ ^75^ ^),(^ ^77^ ^)-(^ ^79^ South, Central, and North America and the Caribbean (k = 12), ^(^ ^42^ ^),(^ ^43^ ^),(^ ^49^ ^),(^ ^59^ ^),(^ ^60^ ^),(^ ^63^ ^),(^ ^65^ ^),(^ ^66^ ^),(^ ^69^ ^),(^ ^70^ ^),(^ ^72^ ^),(^ ^76^ Asia (k = 2), ^(^ ^45^ ^),(^ ^62^ Oceania(k = 2), ^(^ ^53^ ^),(^ ^58^ and Eurasia (k = 1), ^(^ ^41^ using data from about 13,960 men and 2,083 women (Table 1). Regarding the design, the studies were cross-sectional (k = 12) ^(^ ^38^ ^),(^ ^40^ ^)-(^ ^43^ ^),(^ ^50^ ^),(^ ^53^ ^),(^ ^54^ ^),(^ ^59^ ^),(^ ^72^ ^),(^ ^74^ ^),(^ ^76^ or longitudinal (k = 32). ^(^ ^22^ ^),(^ ^35^ ^)-(^ ^37^ ^),(^ ^39^ ^),(^ ^44^ ^)-(^ ^49^ ^),(^ ^51^ ^),(^ ^55^ ^)-(^ ^58^ ^),(^ ^60^ ^),(^ ^62^ ^)-(^ ^71^ ^),(^ ^73^ ^),(^ ^75^ ^),(^ ^77^ ^)-(^ ^79^ Regarding the outcome, the studies provided data on the prevalence of LBP (k = 19), ^(^ ^36^ ^),(^ ^38^ ^)-(^ ^42^ ^),(^ ^45^ ^),(^ ^49^ ^),(^ ^50^ ^),(^ ^53^ ^),(^ ^54^ ^),(^ ^60^ ^),(^ ^62^ ^),(^ ^67^ ^),(^ ^68^ ^),(^ ^72^ ^),(^ ^74^ ^),(^ ^76^ ^),(^ ^78^ the frequency of LBP according to the total number of injuries (k = 34), ^(^ ^22^ ^),(^ ^35^ ^)-(^ ^37^ ^),(^ ^40^ ^),(^ ^42^ ^)-(^ ^49^ ^),(^ ^51^ ^),(^ ^55^ ^)-(^ ^60^ ^),(^ ^63^ ^)-(^ ^71^ ^),(^ ^73^ ^),(^ ^76^ ^)-(^ ^79^ and the incidence of LBP according to 1,000 player-hours of exposure (k = 24). ^(^ ^22^ ^),(^ ^35^ ^),(^ ^36^ ^),(^ ^44^ ^)-(^ ^47^ ^),(^ ^49^ ^),(^ ^51^ ^),(^ ^55^ ^)-(^ ^57^ ^),(^ ^60^ ^),(^ ^63^ ^)-(^ ^66^ ^),(^ ^68^ ^),(^ ^69^ ^),(^ ^71^ ^),(^ ^73^ ^),(^ ^77^ ^)-(^ ^79^

**Table 1:** Characteristics of the studies included in the review (k = 44).

Study Author Date	Location Country(ies)	Design Cross-sectional, longitudinal	Sample Men/women (n)	Assessment Season^*^, period	Exposure Men/women (h) Total^†^	Injury Men/women (n) Total^‡^	Outcome Prevalence^§^, incidence
			Mean age (variability)				
Arnason et al. ^(^ ^36^ 1996	Iceland	Longitudinal	84/0	Apr-Sep/1991, during ≤ 1 season	6,850^¶^/0	85/0	Prevalence, incidence
			25 (18-35) years				
Bjørneboe et al. ^(^ ^37^ 2011	Norway	Longitudinal	296/0	Jul-Nov/2007, during ≤ 1 season	NA	174/0	Prevalence
			NA				
Brynhildsen et al. ^(^ ^38^ 1997	Sweden	Cross-sectional	0/361	At the time of the study (point), lifetime	NA	NA	Prevalence
			21 (14-36) years				
Brynhildsen et al. ^(^ ^39^ 1997	Sweden	Longitudinal	0/261	6-8 months, during ≤ 1 season	NA	NA	Prevalence
			21 (15-28) years				
Cabral^40^ 2017	Portugal	Cross-sectional	48/0	Past year	NA	36/0	Prevalence
			24 (16-38) years				
Çali et al. ^(^ ^41^ 2015	Turkey	Coss-sectional	121/0	Past year	NA	NA	Prevalence
			24 (16-34) years				
Cesca et al. ^(^ ^42^ 2012	Brazil	Cross-sectional	20/0	Jan-May/2011, during ≤ 1 season	NA	58/0	Prevalence
			NA (18-40) years				
Coelho^43^ 2011	Brazil	Cross-sectional	67/0	May-Aug/2011, during ≤ 1 season	NA	66/0	Prevalence
			NA				
Dupont et al. ^(^ ^44^ 2010	Scotland	Longitudinal	32/0	Jul/2007-May/2009, during > 1 season	18,495/0	165/0	Prevalence, incidence
			26 (4) years				
Eirale et al. ^(^ ^45^ 2012	Qatar	Longitudinal	36/0	Jun/2007-Oct/2008, during > 1 season	10,043/0	78/0	Prevalence, incidence
			24 (NA) years				
Ekstrand et al. ^(^ ^46^ 2011	Several^A^	Longitudinal	2,299/0	June/2001-Dec/2009, during > 1 season	1,175,000/0	2,908/0	Prevalence, incidence
			25 (5) years				
Ekstrand et al. ^(^ ^47^ 2011	Several^B^	Longitudinal	613/154	Feb/2003-Oct/2008, during > 1 season	198,071/48,404	1,791/314	Prevalence, incidence
			25 (16-38) years				
			23 (15-38) years				
Ekstrand et al. ^(^ ^22^ 2013	Several^C^	Longitudinal	1,743/0	Jul/2001-Jun/2012, during > 1 season	1,057,201/0	8,029/0	Prevalence, incidence
			NA				
Ekstrand et al. ^(^ ^48^ 2020	Several^D^	Longitudinal	NA/0	2001-2017, during > 1 season	NA	19,926/0	Prevalence
			NA				
Escobar^49^ 2018	Guatemala	Longitudinal	28/0	Jan-Jun/2017, during ≤ 1 season	396^¶^/0	25/0	Prevalence, incidence
			> 20 years				
Grosdent et al. ^(^ ^50^ 2016	Belgium	Cross-sectional	43/0	At the time of the study (point); past year	NA	NA	Prevalence
			18 (1) years				
Hägglund et al. ^(^ ^51^ 2009	Sweden	Longitudinal	239/228	Jan-Oct/2005, during ≤ 1 season	71,361/54,156	548/299	Prevalence, incidence
			25 (16-37) years				
			23 (15-41) years				
Hides et al. ^(^ ^53^ 2016	Australia	Cross-sectional	25/0	At the time of the study (point)	NA	NA	Prevalence
			24 (6) years				
Junge et al. ^(^ ^54^ 2000	Czech Republic	Cross-sectional	81/0	Lifetime	NA	NA	Prevalence
			24 (18-33) years				
Kristenson et al. ^(^ ^55^ 2013	Norway and Sweden	Longitudinal	1,507/0	Jan/2010-Nov/2011, during > 1 season	229,456/0	2,241/0	Prevalence, incidence
			25 (5) years				
Krutsch et al. ^(^ ^56^ 2022	Germany	Longitudinal	1,800^§^/0	Aug/2014-May/2018, during > 1 season	855,000^¶^/0	551/0	Prevalence, incidence
			NA				
Larruskain et al. ^(^ ^57^ 2018	Spain	Longitudinal	50/35	Jul/2010-Jun/2015, during > 1 season	28,878/25,395	323/160	Prevalence, incidence
			25 (4) years				
			25 (5) years				
Lu et al. ^(^ ^58^ 2020	Australia	Longitudinal	421/0	Oct/2012-Apr/2018, during > 1 season	NA	917/0	Prevalence
			NA				
Martín-San Agustín et al. ^(^ ^35^ 2021	Spain	Longitudinal	0/123	Jul/2016-Jun/2017, during ≤ 1 season	0/30,959^¶^	0/113	Prevalence, incidence
			23 (4) years				
Nascimento et al. ^(^ ^59^ 2015	Brazil	Cross-sectional	25/0	Jan-May/2013, during ≤ 1 season	NA	11/0	Prevalence
			24 (4) years				
Netto et al. ^(^ ^60^ 2019	Brazil	Longitudinal	864/0	May-Dec/2016, during ≤ 1 season	12,507^¶^/0	312/0	Prevalence, incidence
			22 (NA) years				
Noormohammadpour et al. ^(^ ^62^ 2020	Iran	Longitudinal	37/0	6 months, during ≤ 1 season	NA	NA	Prevalence
			19 (16-23) years				
Pangrazio et al. ^(^ ^63^ 2016	Several^E^	Longitudinal	506/644	2015-2016, during ≤ 1 season	1,914^¶^/1,584^¶^	115/151	Prevalence, incidence
			NA				
Papacostas et al. ^(^ ^64^ 2009	Greece	Longitudinal	105/0	Jul-May, during > 1 season	11,491/0	51/0	Prevalence, incidence
			26 (5) years				
Paus et al. ^(^ ^65^ 2003	Argentina	Longitudinal	86/0	1995-2001, during > 1 season	3,237/0	2,536/0	Prevalence, incidence
			27 (17-37) years				
Pedrinelli et al. ^(^ ^66^ 2013	Several^F^	Longitudinal	276/0	Jul/2011, during ≤ 1 season	2,430/0	63/0	Prevalence, incidence
			NA				
Peterson et al. ^(^ ^67^ 2000	Czech Republic	Longitudinal	51/0	Past year	NA	99/0	Prevalence
			NA				
Poulsen et al. ^(^ ^68^ 1991	Denmark	Longitudinal	55/0	1986, during ≤ 1 season	6,445/0	57/0	Prevalence, incidence
			26 (21-30) years				
Santos et al. ^(^ ^69^ 2009	Brazil	Longitudinal	35/0	2007, during ≤ 1 season	1,007^¶^/0	49/0	Prevalence, incidence
			NA				
Silva et al. ^(^ ^70^ 2005	Brazil	Longitudinal	30/0	Jan-Dec/2003, during ≤ 1 season	NA	49/0	Prevalence
			NA				
Stubbe et al. ^(^ ^71^ 2015	Netherlands	Longitudinal	217/0	Jul/2009-May/2010, during ≤ 1 season	46,194/0	286/0	Prevalence, incidence
			25 (4) years				
Todeschini et al. ^(^ ^72^ 2019	Brazil	Cross-sectional	39/0	Lifetime	NA	NA	Prevalence
			23 (5) years				
Torrontegui-Duarte et al. ^(^ ^73^ 2020	Spain	Longitudinal	71/0	Aug/1999-May/2017, during > 1 season	50,140^¶^/0	356/0	Prevalence, incidence
			27 (3) years				
Tunås et al. ^(^ ^74^ 2015	Norway	Cross-sectional	0/277	At the time of the study (point); past year; lifetime	NA	NA	Prevalence
			22 (18-32) years				
van Beijsterveldt et al. ^(^ ^75^ ^)#^ 2015	Netherlands	Longitudinal	217/0	Jul/2009-May/2010, during ≤ 1 season	46,194/0	286/0	Prevalence, incidence
			25 (4) years				
Vasconcelos Jr. et al. ^(^ ^76^ 2010	Brazil	Cross-sectional	19/0	May-Nov/2009, during ≤ 1 season	NA	20/0	Prevalence
			27 (4) years				
Waldén et al. ^(^ ^77^ 2005	Several^G^	Longitudinal	266/0	Jul/2001-May/2002, during ≤ 1 season	69,707/0	658/0	Prevalence, incidence
			26 (4) years				
Waldén et al. ^(^ ^78^ 2007	Several^H^	Longitudinal	368/0	Jun-Jul/2004, during ≤ 1 season	4,742/0	45/0	Prevalence, incidence
			NA				
Waldén et al. ^(^ ^79^ 2013	Several^I^	Longitudinal	1,357/0	Aug/2001-May/2010, during > 1 season	773,563/0	5,949/0	Prevalence, incidence
			NA				

n = absolute number; h = hour; NA = not available

*Assessment period in each included study: during ≤ 1 season (≤ 12 months) or > 1 season (> 12 months); or during a given time (e.g., point) and/or period (e.g., past year).

†Total hours of exposure in training and/or matches in each included study.

‡Total soccer-related injuries in each included study.

§Prevalence of LBP according to the total sample (cases/total sample) and/or frequency of LBP according to the total number of injuries (cases/total number of injuries) in each included study.

¶Estimated exposure based on data provided in the included study, in another study with the same sample, and/or in the literature. #This study used data from the same sample as the study by van Stubbe et al. ^(^ ^71^

AData from 51 European teams from several countries such as England, Italy, Germany, Spain, France, the Netherlands, Sweden, among others.

BData from 20 European teams from Sweden, the Netherlands, Finland, Switzerland, Ireland, Norway, Austria, and Scotland.

CData from 27 European teams from 10 countries, such as England, Italy, Netherlands, Spain, Germany, among others.

DData from 116 European teams from 24 countries, such as France, Spain, Germany, Italy, England, Portugal, the Netherlands, Belgium, Norway, Sweden, Switzerland, Denmark, among others. EData from 12 Latin American teams and 16 national teams from Argentina, Brazil, Bolivia, Chile, Colombia, Peru, Ecuador, Paraguay, Uruguay, Venezuela, Costa Rica, Panama, Haiti, Jamaica, Mexico, and the United States.

FInternational tournament with national teams from Argentina, Brazil, Peru, Colombia, Costa Rica, Uruguay, Ecuador, Bolivia, Chile, Venezuela, Mexico, and Paraguay.

GData from 11 European teams from England, France, Italy, Netherlands, and Spain.

HInternational tournament with national teams from Bulgaria, Croatia, Czech Republic, Denmark, England, France, Germany, Greece, Italy, Latvia, Netherlands, Portugal, Russia, Spain, Sweden, and Switzerland.

IData from 24 European teams from Scotland, England, France, Netherlands, Belgium, Germany, Italy, Portugal, and Spain.

### Risk of bias

**Figure 2:**
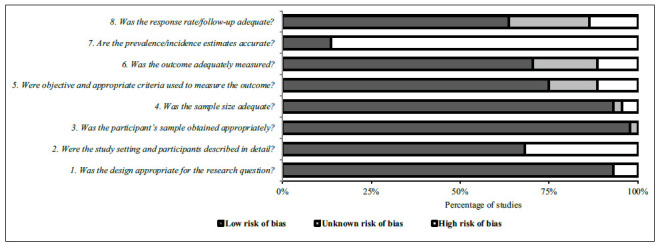
Risk of bias summary of the included studies (*k* = 44).

The assessment of the 44 included studies showed the following results: 93% to 98% of studies had “low risk” in items 1, 3, and 4; 75% and 71% of studies had “low risk” in items 5 and 6, respectively; and 68% and 64% of studies had “low risk” in items 2 and 8, respectively. The main methodological problem was in item 7, as 86% of studies had “high risk,” mainly because they did not provide a CI for prevalence/incidence values (Figure 2). In item 5, which refers to the diagnosis of the condition, 62% of studies (*k* = 27) ^(^ ^22^ ^),(^ ^36^ ^),(^ ^37^ ^),(^ ^44^ ^)-(^ ^48^ ^),(^ ^50^ ^),(^ ^51^ ^),(^ ^55^ ^)-(^ ^58^ ^),(^ ^60^ ^),(^ ^62^ ^),(^ ^64^ ^),(^ ^65^ ^),(^ ^68^ ^)-(^ ^71^ ^),(^ ^73^ ^),(^ ^75^ ^),(^ ^77^ ^)-(^ ^79^ used the definition of soccer-related injury proposed by Fuller et al. ^(^ ^20^ in their consensus statement (i.e., time-loss injury) (Supplementary Table 2). The total average of “low risk” answers was 5.7 (2-8) (Table 2).

**Table 2:** Risk of bias assessment of the included studies (*k* = 44).

Study	Item								
	1	2	3	4	5	6	7	8	Total
	*Study design*	*Setting/ participants*	*Sampling method*	*Sample size*	*Diagnosis*	*Data collection*	*Statistical approach*	*Sample losses*	0-8
Arnason et al. ^(^ ^36^	Y	Y	Y	Y	Y	Y	N	Y	7
Bjørneboe et al. ^(^ ^37^	Y	Y	Y	Y	Y	U	N	Y	6
Brynhildsen et al. ^(^ ^38^	Y	Y	Y	Y	U	Y	N	Y	6
Brynhildsen et al. ^(^ ^39^	Y	Y	Y	Y	Y	N	N	U	5
Cabral^40^	Y	Y	Y	Y	Y	Y	N	Y	7
Çali et al. ^(^ ^41^	Y	Y	Y	Y	N	Y	N	N	5
Cesca et al. ^(^ ^42^	N	Y	Y	N	U	Y	N	Y	4
Coelho^43^	Y	N	Y	Y	N	N	N	Y	4
Dupont et al. ^(^ ^44^	Y	Y	Y	Y	Y	Y	Y	Y	8
Eirale et al. ^(^ ^45^	Y	Y	Y	Y	Y	Y	N	Y	7
Ekstrand et al. ^(^ ^46^	Y	Y	Y	Y	Y	Y	N	U	6
Ekstrand et al. ^(^ ^47^	Y	Y	Y	Y	Y	Y	N	U	6
Ekstrand et al. ^(^ ^22^	Y	N	Y	Y	Y	Y	N	Y	6
Ekstrand et al. ^(^ ^48^	Y	N	Y	U	Y	U	N	U	3
Escobar^49^	Y	N	Y	Y	N	Y	N	Y	5
Grosdent et al. ^(^ ^50^	Y	Y	Y	Y	Y	Y	N	Y	7
Hägglund et al. ^(^ ^51^	Y	Y	Y	Y	Y	Y	N	Y	7
Hides et al. ^(^ ^53^	Y	Y	Y	Y	N	Y	N	Y	6
Junge et al. ^(^ ^54^	Y	Y	Y	Y	U	U	N	Y	5
Kristenson et al. ^(^ ^55^	Y	Y	Y	Y	Y	Y	Y	Y	8
Krutsch et al. ^(^ ^56^	Y	N	Y	Y	Y	Y	N	U	5
Larruskain et al. ^(^ ^57^	Y	Y	Y	Y	Y	Y	Y	Y	8
Lu et al. ^(^ ^58^	Y	N	Y	Y	Y	N	Y	U	5
Martín-San Agustín et al. ^(^ ^35^	Y	Y	Y	Y	Y	Y	Y	N	6
Nascimento et al. ^(^ ^59^	Y	Y	Y	Y	N	U	N	Y	5
Netto et al. ^(^ ^60^	Y	N	Y	Y	Y	Y	N	Y	6
Noormohammadpour et al. ^(^ ^62^	N	Y	Y	Y	Y	U	N	Y	5
Pangrazio et al. ^(^ ^63^	Y	N	Y	Y	U	U	N	Y	4
Papacostas et al. ^(^ ^64^	Y	Y	Y	Y	Y	Y	N	Y	7
Paus et al. ^(^ ^65^	Y	Y	Y	Y	Y	Y	N	U	6
Pedrinelli et al. ^(^ ^66^	Y	N	Y	Y	Y	Y	N	Y	6
Peterson et al. ^(^ ^67^	Y	N	Y	Y	Y	N	N	N	4
Poulsen et al. ^(^ ^68^	Y	Y	Y	Y	Y	Y	N	Y	7
Santos et al. ^(^ ^69^	Y	N	Y	Y	Y	U	N	Y	5
Silva et al. ^(^ ^70^	N	N	U	Y	Y	N	N	U	2
Stubbe et al. ^(^ ^71^	Y	Y	Y	Y	Y	Y	N	N	6
Todeschini et al. ^(^ ^72^	Y	Y	Y	Y	U	Y	N	Y	6
Torrontegui-Duarte et al. ^(^ ^73^	Y	Y	Y	Y	Y	Y	N	N	6
Tunås et al. ^(^ ^74^	Y	Y	Y	Y	Y	Y	N	Y	7
van Beijsterveldt et al. ^(^ ^75^	Y	Y	Y	Y	Y	Y	N	N	6
Vasconcelos Jr. et al. ^(^ ^76^	Y	Y	Y	N	U	U	N	U	3
Waldén et al. ^(^ ^77^	Y	Y	Y	Y	Y	Y	N	Y	7
Waldén et al. ^(^ ^78^	Y	N	Y	Y	Y	Y	N	Y	6
Waldén et al. ^(^ ^79^	Y	N	Y	Y	Y	Y	Y	U	6

Tool developed by Loney and Stratford^18^ and Loney et al. ^(^ ^19^

1. Was the design appropriate for the research question?

2. Were the study setting and participants described in detail?

3. Was the participant’s sample obtained appropriately?

4. Was the sample size adequate?

5. Were objective and appropriate criteria used to measure the outcome?

6. Was the outcome adequately measured?

7. Are the prevalence/incidence estimates accurate?

8. Was the response rate/follow-up adequate?

Y = yes; N = no; U = unclear.

## META-ANALYSES

### Prevalence

**Figure 3:**
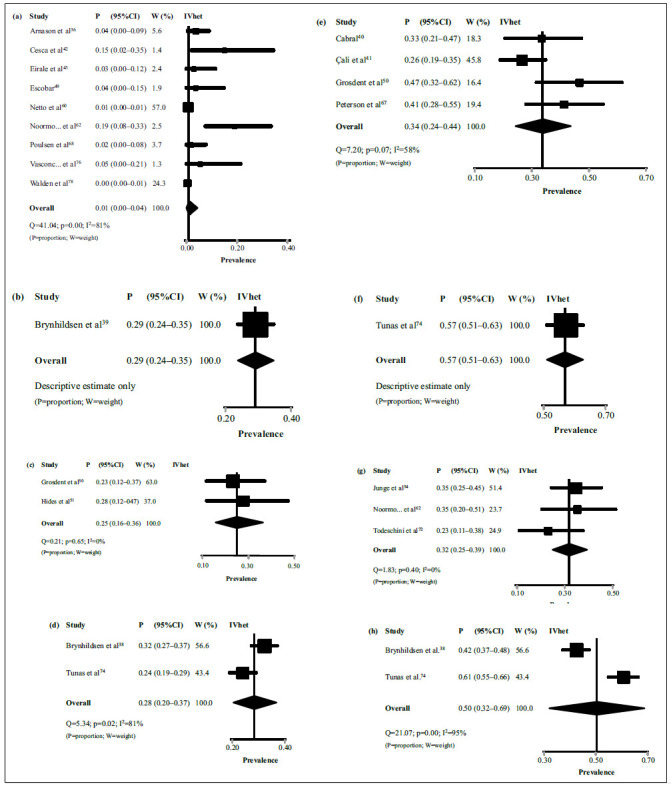
Meta-analyses with pooled prevalence estimates of low back pain in professional soccer players, according to the total number of players, reported in each included study (*k* = 19).

In total, 10 studies^36^ ^),(^ ^39^ ^),(^ ^42^ ^),(^ ^45^ ^),(^ ^49^ ^),(^ ^60^ ^),(^ ^62^ ^),(^ ^68^ ^),(^ ^76^ ^),(^ ^78^ provided the prevalence of LBP during ≤ 1 season (Supplementary Table 3a). The pooled estimate in men was 1% (95%CI = 0-4%) (Figure 3a). The evidence for this estimate was rated as moderate quality due to serious inconsistency (I^2^ = 81%). Descriptively, one study^39^ showed an estimate in women of 29% (95%CI = 24-35%) (Figure 3b).

Four studies^38^ ^),(^ ^50^ ^),(^ ^53^ ^),(^ ^74^ provided the point prevalence of LBP (Supplementary Table 3b). The pooled estimate in men was 25% (95%CI = 16-36%) (Figure 3c). The evidence for this estimate was rated as low quality due to serious risk of bias and indirectness (≥ 50% of pooled studies had “high risk” in item 5 of the risk of bias tool and did not use valid/reliable methods to identify LBP in soccer settings, respectively). The pooled estimate in women was 28% (95%CI = 20-37%) (Figure 3d). The evidence for this estimate was rated as moderate quality due to serious inconsistency (I^2^ = 81%). Five studies^40^ ^),(^ ^41^ ^),(^ ^50^ ^),(^ ^67^ ^),(^ ^74^ provided past-year prevalence of LBP (Supplementary Table 3c). The pooled estimate in men was 34% (95%CI = 24-44%) (Figure 3e). The evidence for this estimate was rated as low quality due to serious risk of bias and indirectness (≥ 50% of pooled studies had “high risk” in items 5 or 6 of the risk of bias tool and did not use valid/reliable methods to identify LBP in soccer settings, respectively). Descriptively, one study^74^ showed an estimate in women of 57% (95%CI = 51-63%) (Figure 3f).

Five studies^38^ ^),(^ ^54^ ^),(^ ^62^ ^),(^ ^72^ ^),(^ ^74^ provided lifetime prevalence of LBP (Supplementary Table 3d). The pooled estimate in men was 32% (95%CI = 25-39%) (Figure 3g). The evidence for this estimate was rated as high quality. The pooled estimate in women was 50% (95%CI = 32-69%) (Figure 3h). The evidence for this estimate was rated as moderate quality due to serious inconsistency (I^2^ = 95%).

### Injury frequency

**Figure 4:**
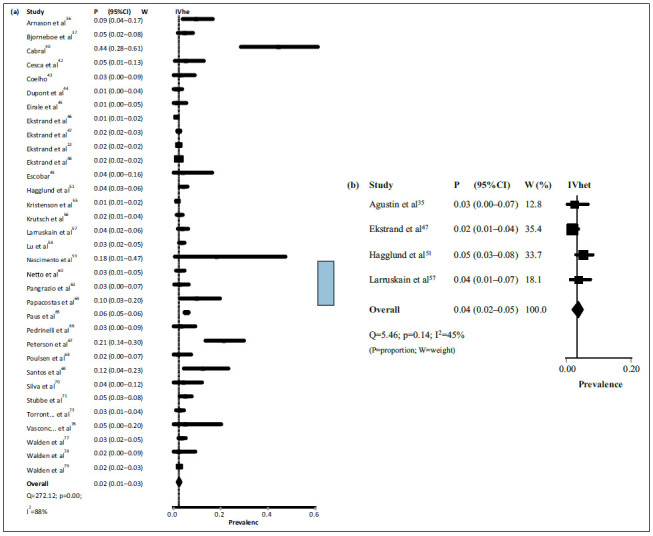
Meta-analyses with pooled frequency estimates of low back pain in professional soccer players, according to the total number of injuries, reported in each included study (*k* = 34).

**Figure 5:**
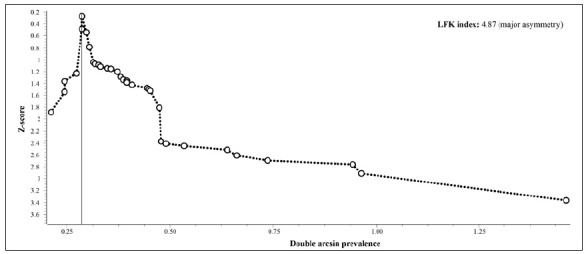
Doi plot of Z-score by double arcsine prevalence (*k* = 33).

In total, 34 studies^22^ ^),(^ ^35^ ^)-(^ ^37^ ^),(^ ^40^ ^),(^ ^42^ ^)-(^ ^49^ ^),(^ ^51^ ^),(^ ^55^ ^)-(^ ^60^ ^),(^ ^63^ ^)-(^ ^71^ ^),(^ ^73^ ^),(^ ^76^ ^)-(^ ^79^ provided the frequency of LBP according to the total number of injuries (Supplementary Table 4). The pooled estimate in men was 2% (95%CI = 1-3%) (Figure 4a). The evidence for this estimate was rated as low quality due to serious inconsistency and publication bias (I^2^ = 88% and presence of “major asymmetry,” with p = 0.02 according to Egger’s test, respectively) (Figure 5). The pooled estimate in women was 4% (95%CI = 2-5%) (Figure 4b). The evidence for this estimate was rated as high quality.

### Incidence

**Figure 6:**
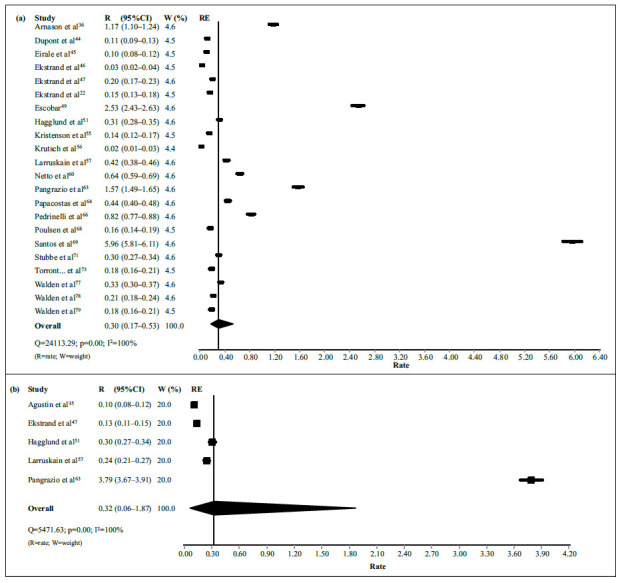
Meta-analyses with pooled incidence rates of low back pain in professional soccer players, according to 1,000 player-hours of exposure, reported in each included study (*k* = 23).

**Figure 7:**
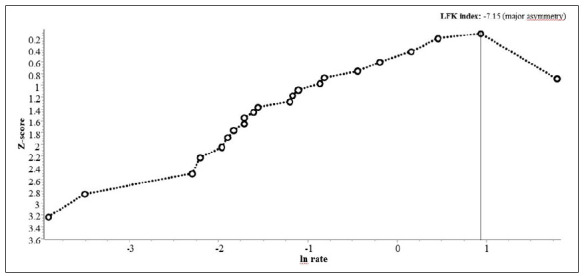
Doi plot of Z-score by rate (*k* = 23).

A total of 24^22^ ^),(^ ^35^ ^),(^ ^36^ ^),(^ ^44^ ^)-(^ ^47^ ^),(^ ^49^ ^),(^ ^51^ ^),(^ ^55^ ^)-(^ ^57^ ^),(^ ^60^ ^),(^ ^63^ ^)-(^ ^66^ ^),(^ ^68^ ^),(^ ^69^ ^),(^ ^71^ ^),(^ ^73^ ^),(^ ^77^ ^)-(^ ^79^ studies provided the incidence of LBP according to 1,000 player-hours of exposure (Supplementary Table 5). The pooled rate in men was 0.30 (95%CI = 0.17-0.53%) (Figure 6a). We excluded one study^65^ from this analysis due to its very extreme rate (43.25; 95%CI = 36.50-50.84). The evidence for this rate was rated as low quality due to serious inconsistency and publication bias (I^2^ = 100% and presence of “major asymmetry,” with p < 0.01 according to Egger’s test, respectively) (Figure 7). The pooled estimate in women was 0.32 (95%CI = 0.06-1.87%) (Figure 6b). The evidence for this estimate was rated as moderate quality due to serious inconsistency (I^2^ = 100%).

### Recurrence, intensity, and severity

Three studies^36^ ^),(^ ^46^ ^),(^ ^48^ provided the recurrence rate of LBP (only in men), which ranged from 3% to 63%. Five studies^40^ ^),(^ ^41^ ^),(^ ^50^ ^),(^ ^53^ ^),(^ ^62^ provided the intensity of LBP, which ranged from 1.68 (2.39) to 4.87 (2.14) points on a 0-10 scale. Three of these studies^40^ ^),(^ ^41^ ^),(^ ^50^ reported a minimum of 0 and a maximum of 8 points. Five studies^22^ ^),(^ ^45^ ^),(^ ^46^ ^),(^ ^48^ ^),(^ ^57^ provided the days a player is absent from professional activities due to pain, which ranged from 2 (0) to 10 (19) days. Four of these studies^45^ ^),(^ ^46^ ^),(^ ^48^ ^),(^ ^57^ reported a minimum of one and a maximum of 28 days absent.

## DISCUSSION

### General findings

This review included 44 original studies with epidemiological (prevalence and incidence) and clinical (recurrence and severity) data on LBP in professional soccer players. Most studies scored “low risk” in the assessment of bias. Meta-analyses of the prevalence, frequency (according to the total number of injuries), and incidence of LBP provided pooled estimates with quality of evidence ranging from high to low according to the GRADE system. Few studies reported data on the recurrence, intensity, and severity of LBP, with considerable variation between results.

### Prevalence findings

The prevalence of LBP in men showed a consistent increase as the exposure/assessment time of the original studies increased. The estimate (1%) was lower when pooling studies that evaluated LBP during ≤ 1 season (e.g., tournaments and championships), but higher (34%) when pooling studies that assessed LBP in the past year. In fact, longer exposure/assessment periods are more sensitive in capturing positive cases, especially for conditions that may present short-term signs/symptoms, such as an acute episode of LBP. ^(^ ^18^ Other reviews on the epidemiology of LBP in professional sports also show this same pattern of prevalence estimates. ^(^ ^4^ ^),(^ ^5^ ^),(^ ^80^ On the other hand, for women, the inconsistency between prevalence estimates was greater, since only one study provided estimates during ≤ 1 season (29%) and in the past year (57%). Despite this, point prevalence was consistently lower (28%) compared with lifetime prevalence (50%).

### Injury frequency findings

The frequency of LBP according to the total number of injuries showed 1,165 events/48,577 injuries (2%) in men and 31 events/886 injuries (4%) in women. Recent longitudinal studies using a similar definition of soccer-related injury (time-loss injury) also show estimates of LBP from 2% to 2.5% in men^48^ ^),(^ ^56^ ^),(^ ^73^ and from 2.7% to 3.8% in women, ^(^ ^35^ ^),(^ ^57^ ^),(^ ^63^ while older studies report estimates above 5%.^51^ ^),(^ ^81^ Over the past few years, new preventive approaches implemented within professional soccer, such as the identification of potential risk factors, the improvement of specialized medical practices, and individualized care, may have contributed to the reduction in the estimates of LBP. ^(^ ^6^ ^),(^ ^80^ ^),(^ ^81^ Moreover, other aspects related to soccer itself such as the player’s position on the field, can have a significant effect on back complaints. For example, Onaka et al. ^(^ ^82^ found a wide variation in the occurrence of LBP according to field position compared with other conditions (e.g., groin pain). Forwards (4.1%) and defensive midfielders (5.2%) had lower estimates, while goalkeepers (28.6) and attacking midfielders (43.1%) had higher estimates. Differences in the biomechanical demands of the musculoskeletal system depending on field position may explain this variation in estimates. ^(^ ^82^

### Incidence findings

The incidence of LBP per 1,000 player-hours of exposure showed similar rates in men (0.30) and women (0.32). However, the pooled rate in women shows a wide CI range compared with the pooled rate in men, which may be attributed to the small number of included studies evaluating the incidence of LBP in female soccer players (*k* = 5). These rates corroborate the high epidemiological burden of LBP among soccer-related injuries worldwide. ^(^ ^4^ ^),(^ ^6^ A recent systematic review on the epidemiology of injuries in professional soccer settings showed that the rate of injuries affecting the trunk region (e.g., spine) was 0.40 per 1,000 player-hours of exposure, making it the second most affected anatomical site after lower limb injuries. ^(^ ^32^ LBP contributes to most injuries that affect the trunk in professional soccer players, as evidenced by several primary studies. ^(^ ^35^ ^),(^ ^44^ ^),(^ ^47^ ^),(^ ^57^ ^),(^ ^66^ ^),(^ ^69^ ^),(^ ^71^ ^),(^ ^79^ Compared with other elite sports, the incidence rate of LBP is higher in basketball (0.40/1,000 hours of exposure), ^(^ ^83^ mainly due to a combination of factors, such as overload and trauma to the lumbar region, ^(^ ^6^ and in rowing (1.67/1,000 hours of exposure), ^(^ ^84^ mainly due to the exacerbated increase in tension in the lumbar paraspinal muscles. ^(^ ^5^

### Recurrence, intensity, and severity findings

A very small number of studies reported the recurrence of LBP (*k* = 3). Two of these studies evaluated large samples over long follow-up periods (Ekstrand, Hägglund, and Waldén, ^(^ ^46^ *n* ≅ 2,299, 1-9 seasons; and Ekstrand et al., ^(^ ^48^ *n* ≅ 12,350, 1-16 seasons) and provided recurrence rates of 3 and 18.8%, respectively. Although previous guidelines presented recommendations for assessing injury recurrence in soccer (e.g., definition and use), ^(^ ^20^ ^),(^ ^23^ this measure has not been used in most epidemiological studies, thus failing to show the burden of injury recurrence in professional players. Four original studies reported the intensity of LBP (0-10), which ranged from mild (0-3) to moderate (4-7). Maintaining adequate physical condition, flexibility, and muscle strength of the trunk and lower limbs can be a protective factor against severe injuries that manifest with higher pain intensity. ^(^ ^9^ ^),(^ ^50^ Hides et al. ^(^ ^53^ found that additional muscle training programs (e.g., strengthening) performed by players during the pre-season to prevent injuries was associated with a significant increase in the cross-sectional area of the multifidus muscle and a clinically important decrease in pain intensity in players suffering from LBP at baseline. Five original studies reported the severity of LBP (days absent from professional activities due to pain), which ranged from one to 28 days (average of 2 [0] to 10 [19] days). This finding highlights that most players with LBP had a severity ranging from minimal (≤ 3 days) to moderate (8-28 days), ^(^ ^20^ which suggests the presence of an acute condition (≤ 6 weeks). ^(^ ^15^

### Practical implications

Although the included studies provided good data on the occurrence of LBP, this condition is still poorly studied as a primary outcome in professional soccer. Much of the literature specifically on back pain in soccer includes male, young, and non-elite athletes. ^(^ ^6^ ^),(^ ^7^ ^),(^ ^85^ The results of this review showed that the epidemiological burden of LBP in professional players may be significant in men (prevalence of 1% to 34%), but consistently higher in women (prevalence of 28% to 57%). Considering both sexes, at least one in four players is likely to suffer from LBP at any given time. With an ever-increasing level of physical demand, health professionals who treat professional players should be alert to the causal mechanisms of lumbar injuries, including acute/traumatic (e.g., muscle strains, trunk hyperextension/hyperflexion, direct contusions, and sitting falls) and chronic/overuse (e.g., repetitive stress, microtraumas, overload, and degenerative changes). ^(^ ^6^ ^),(^ ^9^ Particularly in women, a U-shaped perspective should also be considered, since low or (conversely) strenuous levels of sport activities are associated with LBP. ^(^ ^86^ Moreover, other aspects, such as less pre-season physical conditioning, the large number of matches played as a starter, and field position can significantly increase estimates of LBP. ^(^ ^41^ ^),(^ ^53^ ^),(^ ^74^ ^),(^ ^82^ All these factors are relevant for preventive efforts in clinical practice.

### Potential limitations

This was a large-scale literature review, with extensive search, inclusion, and analysis of data on the epidemiology of LBP in professional soccer players. The potential limitations of the review add to the limitations of the existing literature on this topic: (a) the different definitions of LBP as an injury in soccer settings (e.g., pain with or without restriction of sports practice; need or not for medical care; and time-loss injury) are a potential source of important heterogeneity, which may have contributed to inconsistencies in some meta-analyses; (b) most of the included studies did not assess LBP as a primary outcome, which limited the acquisition of additional data and secondary analyses (e.g., age group and field position); (c) we did not estimate the prevalence during ≤ 1 season and in the past year in women, and the recurrence, intensity, and severity of LBP due to the insufficient number of included studies (*k* = 1) and/or the very wide variation between results. Future studies assessing back pain in soccer settings should address these limitations.

## CONCLUSION

To the best of our knowledge, this is the first review to evaluate the epidemiology of LBP in professional soccer players. For men, high-quality evidence corresponds to a lifetime prevalence of 32%; moderate-quality evidence corresponds to a prevalence during ≤ 1 season of 1%; and low-quality evidence corresponds to a point prevalence of 25%, a prevalence in the past year of 34%, a frequency (according to the total number of injuries) of 2%; and an incidence rate of 0.30 per 1,000 player-hours of exposure. For women, high-quality evidence refers to a frequency (according to the total number of injuries) of 4%; and moderate-quality evidence refers to a point prevalence of 28%, a lifetime prevalence of 50%, and an incidence rate of 0.32 per 1,000 player-hours of exposure. These results can be used by sports clubs, medical teams, and/or athletes to develop preventive and management strategies aimed at reducing the occurrence of LBP in elite soccer.

## SUPPLEMENTARY MATERIAL

**Table 1:** Search strategies performed on April 6, 2021.

**Embase**
(((backache:ti,ab,kw OR ‘low back pain':ti,ab,kw) AND sport:ti,ab,kw OR injury:ti,ab,kw OR ‘sport injury':ti,ab,kw) AND football:ti,ab,kw OR soccer:ti,ab,kw) AND athlete:ti,ab,kw
**LILACS**
(“back pain”) OR (“low back pain”) OR (backache) OR (lumbago) OR (spine) AND (injury) OR (“sport injury”)
OR (“sports injuries”) AND (football) OR (soccer) OR (athletes) OR (players)
**PubMed/MEDLINE**
(((((((“Epidemiology”[Mesh] OR “epidemiology” [Subheading]) OR ( “Prevalence”[Mesh] OR “Cross-Sectional Studies”[Mesh] )) OR ( “Incidence”[Mesh]
OR “Cohort Studies”[Mesh] )) AND “Back Pain”[Mesh]) OR “Low Back Pain”[Mesh]) OR “Back Injuries”[Mesh]) AND ( “Football/injuries”[Mesh]
OR “Football/statistics and numerical data”[Mesh] )) OR ( “Soccer/injuries”[Mesh] OR “Soccer/statistics and numerical data”[Mesh])
**SciELO**
(“back pain”) OR (“low back pain”) OR (backache) OR (lumbago) OR (spine) OR (injury) OR (“sport injury”)
OR (“sports injuries”) AND (football) OR (soccer) OR (athletes) OR (players)
**Scopus**
TITLE-ABS-KEY ( “back pain” OR “low back pain” OR “back injury” OR “lumbar pain” OR backache OR lumbago OR “spinal pain” AND sport AND football OR soccer OR athletes OR players OR professionals OR elite)
**SPORTDiscus**
(back pain or low back pain or lumbar pain or spinal pain or backache or lumbago or back injury) AND ( players or athletes or professionals or elite ) AND ( sport or football or soccer or ball )
**Web of Science**
#1 TS = (back pain OR low back pain OR back injury OR lumbar pain OR backache OR lumbago OR spinal pain)
#2 TS = (players OR athletes OR professionals OR elite)
#3 TS = (sports OR football OR soccer)
#4 #3 AND #2 AND #1

**Table 2:** Definitions of soccer-related injury used in the included studies (k = 44).

Study	Definition	Reference
Arnason et al. ^(^ ^36^	Unable to participate in a match or training session because of an injury incurred in soccer (time-loss injury).	Lewin^87^
Bjørneboe et al. ^(^ ^37^	Unable to take full part in football activity or match play at least 1 day beyond the day of injury (time-loss injury).	Fuller et al. ^(^ ^20^
Brynhildsen et al. ^(^ ^38^	Woman's subjective feeling of back pain.	NA
Brynhildsen et al. ^(^ ^39^	Experience of back pain during the last active soccer playing season but did not have to prevent the woman from her daily activities or from taking part in practice sessions or games.	NA
Cabral^40^	Pain, ache, or discomfort in the lower back with or without radiation to one or both legs.	Kuorinka et al. ^(^ ^88^
Çali et al. ^(^ ^41^	NA	NA
Cesca et al. ^(^ ^42^	NA	NA
Coelho^43^	NA	NA
Dupont et al. ^(^ ^44^	According to Fuller et al. ^(^ ^20^ (time-loss injury).	Fuller et al. ^(^ ^20^
Eirale et al. ^(^ ^45^	According to Fuller et al. ^(^ ^20^ (time-loss injury).	Fuller et al. ^(^ ^20^
Ekstrand et al. ^(^ ^46^	Traumatic distraction or overuse injury to the muscle, leading to a player being unable to fully participate in training or match play (time-loss injury).	Ekstrand et al. ^(^ ^46^
Ekstrand et al. ^(^ ^47^	According to Fuller et al. ^(^ ^20^ (time-loss injury).	Fuller et al. ^(^ ^20^
Ekstrand et al. ^(^ ^22^	According to Fuller et al. ^(^ ^20^ (time-loss injury).	Fuller et al. ^(^ ^20^
Ekstrand et al. ^(^ ^48^	According to Fuller et al. ^(^ ^20^ (time-loss injury).	Fuller et al. ^(^ ^20^
Escobar^49^	NA	NA
Grosdent et al. ^(^ ^50^	Any physical complaint that is the result of participating in football training or a football match, leading to a player being unable to fully participate in future football training or match play (time-loss injury).	NA
Hägglund et al. ^(^ ^51^	Physical complaint resulting from football training or match play, leading to the player being unable to participate fully in at least one training session or match (time-loss injury).	Hägglund et al. ^(^ ^23^
Hides et al. ^(^ ^53^	Pain localized between T12 and the gluteal fold.	Hides et al. ^(^ ^89^
Junge et al. ^(^ ^54^	NA	NA
Kristenson et al. ^(^ ^55^	According to Fuller et al. ^(^ ^20^ (time-loss injury).	Fuller et al. ^(^ ^20^
Krutsch et al. ^(^ ^56^	Absence from official football matches of at least 28 days (severe injury).	Fuller et al. ^(^ ^20^
Larruskain et al. ^(^ ^57^	According to Fuller et al. ^(^ ^20^ (time-loss injury).	Fuller et al. ^(^ ^20^
Lu et al. ^(^ ^58^	According to Fuller et al. ^(^ ^20^ (time-loss injury).	Fuller et al. ^(^ ^20^
Martín-San Agustín et al. ^(^ ^35^	Any physical complaint sustained by a player that results from a soccer match or training, irrespective of the need for medical attention.	Fuller et al. ^(^ ^20^
Nascimento et al. ^(^ ^59^	NA	NA
Netto et al. ^(^ ^60^	According to Fuller et al. ^(^ ^20^ (time-loss injury).	Fuller et al. ^(^ ^20^
Noormohammadpour et al. ^(^ ^62^	Pain between the last rib and the lower gluteal fold, which is bad enough to limit or change athletes' daily routine or sports activities for more than 1 day (time-loss injury).	Noormohammadpour^90^
Pangrazio et al. ^(^ ^63^	NA	NA
Papacostas et al. ^(^ ^64^	Any mishap occurring during scheduled games or practices that causes a player to miss a subsequent game or practice session (time-loss injury).	Nicholas and Hershman^91^
Paus et al. ^(^ ^65^	An injury occurring during soccer practice, which caused the athlete to miss training and games, followed by the need for anatomical diagnosis of the injured tissue and corresponding treatment (time-loss injury)”.	Dvorak and Junge^92^
Pedrinelli et al. ^(^ ^66^	Any physical complaint sustained by a player that results from a football match or football training, irrespective of the need for medical attention or time-loss from activities.	Fuller et al. ^(^ ^20^
Peterson et al. ^(^ ^67^	Any tissue damage caused by football regardless of the consequences with respect to absence from training or match.	Junge and Dvorak^93^
Poulsen et al. ^(^ ^68^	Any injury occurring during scheduled games or practices which caused the player to miss the next game or practice session (time-loss injury).	Ekstrand^94^
Santos et al. ^(^ ^69^	Absence of the athletes from their professional activities for at least 48 hours (time-loss injury).	NA
Silva et al. ^(^ ^70^	Any event that occurs during games or training of the club, with a reduction or complete absence from the participation of athletes in their sports activities (time-loss injury).	Schmidt-Olsen et al. ^(^ ^95^
Stubbe et al. ^(^ ^71^	According to Fuller et al. ^(^ ^20^ (time-loss injury).	Fuller et al. ^(^ ^20^
Todeschini et al. ^(^ ^72^	NA	NA
Torrontegui-Duarte et al. ^(^ ^73^	Musculoskeletal complaint (pain and/or discomfort) reported by players to the medical staff and receiving medical attention (medical-attention injury), and injuries resulting in a player being unable to fully participate in future training or match play (time-loss injury).	Fuller et al. ^(^ ^20^
Tunås et al. ^(^ ^74^	Pain, ache, or discomfort in the lower back with or without radiation to one or both legs.	Kuorinka et al. ^(^ ^88^
van Beijsterveldt et al. ^(^ ^75^	According to Fuller et al. ^(^ ^20^ (time-loss injury).	Fuller et al. ^(^ ^20^
Vasconcelos Jr. et al. ^(^ ^76^	NA	NA
Waldén et al. ^(^ ^77^	According to Ekstrand^94^ (time-loss injury).	Ekstrand^94^
Waldén et al. ^(^ ^78^	According to Hägglund et al. ^(^ ^23^ (time-loss injury).	Hägglund et al. ^(^ ^23^
Waldén et al. ^(^ ^79^	According to Hägglund et al. ^(^ ^23^ (time-loss injury).	Hägglund et al. ^(^ ^23^

NA = not available

**Table 3:** Prevalence estimates of low back pain in professional soccer players, according to the total number of players, reported in each included study (k = 19).

Study	Prevalence					
Author	Men			Women		
	n	%	95%CI	n	%	95%CI
***a) During* ≤ *1 season/*≤ *12 months (k = 10)***						
Arnason et al. ^(^ ^36^	3	3.6	0.8-10.4	-	-	-
Brynhildsen et al. ^(^ ^39^	-		-	76	29.1	23.9-34.9
Cesca et al. ^(^ ^42^	3	15.0	5.2-36.0	-	-	-
Eirale et al. ^(^ ^45^	1	2.8	0.5-14.2	-	-	-
Escobar^49^	1	3.6	0.6-17.7	-	-	-
Netto et al. ^(^ ^60^	5	0.6	0.2-1.4	-	-	-
Noormohammadpour et al. ^(^ ^62^	7	18.9	9.5-34.2	-	-	-
Poulsen et al. ^(^ ^68^	1	1.8	-0.6-10.5	-	-	-
Vasconcelos Jr. et al. ^(^ ^76^	1	5.3	0.9-24.6	-	-	-
Waldén et al. ^(^ ^78^	1	0.3	-0.1-1.7	-	-	-
***b) Point prevalence (k = 4)***						
Brynhildsen et al. ^(^ ^38^	-	-	-	116	32.1	27.5-37.1
Grosdent et al. ^(^ ^50^	10	23.3	13.0-37.9	-	-	-
Hides et al. ^(^ ^53^	7	28.0	14.3-47.6	-	-	-
Tunås et al. ^(^ ^74^	-	-	-	66	24.1	19.5-29.7
*c) Past-year prevalence (k = 5)*						
Cabral^40^	16	33.3	21.6-47.5	-	-	-
Çali et al. ^(^ ^41^	32	31.4	23.8-40.2	-	-	-
Grosdent et al. ^(^ ^50^	20	43.5	32.5-61.1	-	-	-
Peterson et al. ^(^ ^67^	21	41.2	28.7-54.9	-	-	-
Tunås et al. ^(^ ^74^	-	-	-	158	56.9	51.1-62.7
**d) Lifetime prevalence (k = 5)**						
Brynhildsen et al. ^(^ ^38^	-	-	-	153	42.4	37.4-47.5
Junge et al. ^(^ ^54^	28	34.6	25.1-45.4	-	-	-
Noormohammadpour et al. ^(^ ^62^	13	35.1	21.8-51.2	-	-	-
Todeschini et al. ^(^ ^72^	9	23.1	12.7-38.3	-	-	-
Tunås et al. ^(^ ^74^	-	-	-	168	60.6	54.7-66.2

n = absolute number of players with low back pain; % = prevalence; 95% CI = 95% confidence interval

**Table 4:** Frequency estimates of low back pain in professional soccer players, according to the total number of injuries, reported in each included study (k = 34).

Study	Frequency					
Author	Men			Women		
	n	%	95%CI	n	%	95%CI
Arnason et al. ^(^ ^36^	8	9.4	4.6-17.7	-	-	-
Bjørneboe et al. ^(^ ^37^	8	4.6	2.2-9.0	-	-	-
Cabral^40^	16	33.3	21.6-47.1	-	-	-
Cesca et al. ^(^ ^42^	3	5.2	1.2-14.7	-	-	-
Coelho^43^	2	3.0	0.2-11.0	-	-	-
Dupont et al. ^(^ ^44^	2	1.2	0.1-4.6	-	-	-
Eirale et al. ^(^ ^45^	1	1.3	-0.5-7.6	-	-	-
Ekstrand et al. ^(^ ^46^	32	1.1	0.8-1.6	-	-	-
Ekstrand et al. ^(^ ^47^	39	2.2	1.6-3.0	6	1.9	0.8-4.2
Ekstrand et al. ^(^ ^22^	163	2.0	1.7-2.4	-	-	-
Ekstrand et al. ^(^ ^48^	405	2.0	1.9-2.2	-	-	-
Escobar^49^	1	4.0	0.7-19.5	-	-	-
Hägglund et al. ^(^ ^51^	22	4.0	2.6-6.0	16	5.4	3.3-8.6
Kristenson et al. ^(^ ^55^	33	1.5	1.0-2.1	-	-	-
Krutsch et al. ^(^ ^56^	13	2.4	1.3-4.0	-	-	-
Larruskain et al. ^(^ ^57^	12	3.7	2.1-6.5	6	3.8	1.6-8.1
Lu et al. ^(^ ^58^	31	3.4	2.4-4.8	-	-	-
Martín-San Agustín et al. ^(^ ^35^	-	-	-	3	2.7	0.6-7.9
Nascimento et al. ^(^ ^59^	2	18.2	5.1-47.7	-	-	-
Netto et al. ^(^ ^60^	8	2.6	1.2-5.1	-	-	-
Pangrazio et al. ^(^ ^63^	3	2.6	0.6-7.2	6	3.4	1.7-8.6
Papacostas et al. ^(^ ^64^	5	9.8	3.8-21.4	-	-	-
Paus et al. ^(^ ^65^	140	5.8	4.9-6.7	-	-	-
Pedrinelli et al. ^(^ ^66^	2	3.2	0.2-11.5	-	-	-
Peterson et al. ^(^ ^67^	21	41.2	28.7-54.9	-	-	-
Poulsen et al. ^(^ ^68^	1	1.8	-0.6-10.2	-	-	-
Santos et al. ^(^ ^69^	6	12.2	5.4-24.6	-	-	-
Silva et al. ^(^ ^70^	2	4.1	0.4-14.5	-	-	-
Stubbe et al. ^(^ ^71^ ^)*^	14	4.9	2.9-8.1	-	-	-
Torrontegui-Duarte et al. ^(^ ^73^	9	2.5	1.3-4.8	-	-	-
Vasconcelos Jr. et al. ^(^ ^76^	1	5.0	0.9-23.6	-	-	-
Waldén et al. ^(^ ^77^	23	3.5	2.3-5.2	-	-	-
Waldén et al. ^(^ ^78^	1	2.2	-0.7-12.6	-	-	-
Waldén et al. ^(^ ^79^	136	2.3	1.9-2.7	-	-	-

n = absolute number of players with low back pain; % = frequency; 95% CI = 95% confidence interval

*This study used data from the same sample as the study by van Beijsterveldt et al. ^(^ ^75^

**Table 5:** Incidence rates of low back pain in professional soccer players, according to 1,000 player-hours of exposure, reported in each included study (k = 24).

Study	Incidence			
Author	Men		Women	
	R	95%CI	R	95%CI
Arnason et al. ^(^ ^36^	1.17	0.50-2.30	-	-
Dupont et al. ^(^ ^44^	0.11	0.01-0.39	-	-
Eirale et al. ^(^ ^45^	0.10	0.00-0.56	-	-
Ekstrand et al. ^(^ ^46^	0.03	0.02-0.04	-	-
Ekstrand et al. ^(^ ^47^	0.20	0.14-0.27	0.13	0.05-0.27
Ekstrand et al. ^(^ ^22^	0.15	0.13-0.18	-	-
Escobar^49^	2.53	0.06-14.07	-	-
Hägglund et al. ^(^ ^51^	0.31	0.19-0.47	0.30	0.17-0.48
Kristenson et al. ^(^ ^55^	0.14	0.10-0.20	-	-
Krutsch et al. ^(^ ^56^	0.02	0.01-0.03	-	-
Larruskain et al. ^(^ ^57^	0.42	0.21-0.73	0.24	0.09-0.51
Martín-San Agustín et al. ^(^ ^35^	-	-	0.10	0.02-0.26
Netto et al. ^(^ ^60^	0.64	0.28-1.26	-	-
Pangrazio et al. ^(^ ^63^	1.57	0.32-4.57	3.79	1.39-8.23
Papacostas et al. ^(^ ^64^	0.44	0.14-1.02	-	-
Paus et al. ^(^ ^65^	43.25	36.50-50.84	-	-
Pedrinelli et al. ^(^ ^66^	0.82	0.10-2.97	-	-
Poulsen et al. ^(^ ^68^	0.16	0.00-0.86	-	-
Santos et al. ^(^ ^69^	5.96	2.19-12.92	-	-
Stubbe et al. ^(^ ^71^ ^)*^	0.30	0.17-0.51	-	-
Torrontegui-Duarte et al. ^(^ ^73^	0.18	0.08-0.34	-	-
Waldén et al. ^(^ ^77^	0.33	0.21-0.50	-	-
Waldén et al. ^(^ ^78^	0.21	0.00-1.17	-	-
Waldén et al. ^(^ ^79^	0.18	0.15-0.21	-	-

R = rate; 95% CI = 95% confidence interval

*This study used data from the same sample as the study by van Beijstervel+dt et al. ^(^ ^75^

## References

[1] Wu A, March L, Zheng X, Huang J, Wang X, Zhao J (2020). Global low back pain prevalence and years lived with disability from 1990 to 2017: estimates from the Global Burden of Disease Study 2017. Ann Transl Med.

[2] Carregaro RL, Tottoli CR, Rodrigues DS, Bosmans JE, Silva EN, van Tulder M (2020). Low back pain should be considered a health and research priority in Brazil lost productivity and healthcare costs between 2012 to 2016. PLoS One.

[3] O'Sullivan P, Caneiro JP, O'Keeffe M, O'Sullivan K (2016). Unraveling the complexity of low back pain. J Orthop Sports Phys Ther.

[4] Trompeter K, Fett D, Platen P (2017). Prevalence of back pain in sports a systematic review of the literature. Sports Med.

[5] Farahbakhsh F, Rostami M, Noormohammadpour P, Mehraki Zade A, Hassanmirazaei B, Faghih Jouibari M (2018). Prevalence of low back pain among athletes a systematic review. J Back Musculoskelet Rehabil.

[6] Ball JR, Harris CB, Lee J, Vives MJ (2019). Lumbar spine injuries in sports review of the literature and current treatment recommendations. Sports Med Open.

[7] Mortazavi J, Zebardast J, Mirzashahi B (2015). Low back pain in athletes. Asian J Sports Med.

[8] Eliakim E, Morgulev E, Lidor R, Meckel Y (2020). Estimation of injury costs financial damage of English Premier League teams' underachievement due to injuries. BMJ Open Sport Exerc Med.

[9] Plais N, Salzmann SN, Shue J, Sanchez CD, Urraza FJ, Girardi FP (2019). Spine injuries in soccer. Curr Sports Med Rep.

[10] Munn Z, Moola S, Lisy K, Riitano D, Tufanaru C, Aromataris E, Munn Z (2020). JBI manual for evidence synthesis.

[11] Stroup DF, Berlin JA, Morton SC, Olkin I, Williamson GD, Rennie D (2000). Meta-analysis of observational studies in epidemiology a proposal for reporting. JAMA.

[12] Higgins J, Thomas J, Chandler J, Cumpston M, Li T, Page M (2021). Cochrane Handbook for Systematic Reviews of Interventions, version 6.2.

[13] Page MJ, McKenzie JE, Bossuyt PM, Boutron I, Hoffmann TC, Mulrow CD (2021). The PRISMA 2020 statement an updated guideline for reporting systematic reviews. BMJ.

[14] Ardern CL, Büttner F, Andrade R, Weir A, Ashe MC, Holden S (2022). Implementing the 27 PRISMA 2020 Statement items for systematic reviews in the sport and exercise medicine, musculoskeletal rehabilitation and sports science fields the PERSiST (implementing Prisma in Exercise, Rehabilitation, Sport medicine and SporTs science) guidance. Br J Sports Med.

[15] Malliou P, Gioftsidou A, Beneka A, Godolias G (2006). Measurements and evaluations in low back pain patients. Scand J Med Sci Sports.

[16] Hoskins W, Sakai Y (2012). Low back pain pathogenesis and treatment.

[17] Dvorak J, Graf-Baumann T, Peterson L, Junge A (2000). Football, or soccer, as it is called in North America, is the most popular sport worldwide. Am J Sports Med.

[18] Loney PL, Stratford PW (1999). The prevalence of low back pain in adults a methodological review of the literature. Phys Ther.

[19] Loney PL, Chambers LW, Bennett KJ, Roberts JG, Stratford PW (1998). Critical appraisal of the health research literature prevalence or incidence of a health problem. Chronic Dis Can.

[20] Fuller CW, Ekstrand J, Junge A, Andersen TE, Bahr R, Dvorak J (2006). Consensus statement on injury definitions and data collection procedures in studies of football (soccer) injuries. Br J Sports Med.

[21] Soares AJG, Melo LBS, Costa FR, Bartholo TL, Bento JO (2011). Relationship between formation of young players in Brazil and education. Rev Bras Cienc Esporte.

[22] Ekstrand J, Hägglund M, Kristenson K, Magnusson H, Waldén M (2013). Fewer ligament injuries but no preventive effect on muscle injuries and severe injuries an 11-year follow-up of the UEFA Champions League injury study. Br J Sports Med.

[23] Hägglund M, Waldén M, Bahr R, Ekstrand J (2005). Methods for epidemiological study of injuries to professional football players developing the UEFA model. Br J Sports Med.

[24] Timpka T, Jacobsson J, Bickenbach J, Finch CF, Ekberg J, Nordenfelt L (2014). What is a sports injury. Sports Med.

[25] Lazcano G, Papuzinski C, Madrid E, Arancibia M (2019). General concepts in biostatistics and clinical epidemiology observational studies with cohort design. Medwave.

[26] Hespanhol LC, Barboza SD, van Mechelen W, Verhagen E (2015). Measuring sports injuries on the pitch a guide to use in practice. Braz J Phys Ther.

[27] Brown LD, Cai TT, DasGupta A (2001). Interval estimation for a binomial proportion. Stat Sci.

[28] Doi SA, Barendregt JJ, Khan S, Thalib L, Williams GM (2015). Advances in the meta-analysis of heterogeneous clinical trials I the inverse variance heterogeneity model. Contemp Clin Trials.

[29] Doi SAR, Furuya-Kanamori L, Thalib L, Barendregt JJ (2017). Meta-analysis in evidence-based healthcare a paradigm shift away from random effects is overdue. Int J Evid Based Healthc.

[30] DerSimonian R, Laird N (1986). Meta-analysis in clinical trials. Control Clin Trials.

[31] Barendregt JJ, Doi SAR, Lee YY, Norman RE, Vos T (2013). Meta-analysis of prevalence. J Epidemiol Community Health.

[32] López-Valenciano A, Ruiz-Pérez I, Garcia-Gómez A, Vera-Garcia FJ, De Ste Croix M, Myer GD, Ayala F (2020). Epidemiology of injuries in professional football a systematic review and meta-analysis. Br J Sports Med.

[33] Furuya-Kanamori L, Barendregt JJ, Doi SAR (2018). A new improved graphical and quantitative method for detecting bias in meta-analysis. Int J Evid Based Healthc.

[34] Schünemann H, Brozek J, Guyatt G, Oxman A (2013). GRADE Handbook. Grading the quality of evidence and the strength of recommendations using the GRADE approach (updated October 2013).

[35] Martín-San Agustín RS, Medina-Mirapeix F, Esteban-Catalán A, Escriche-Escuder A, Sánchez-Barbadora M, Benítez-Martínez JC (2021). Epidemiology of injuries in first division Spanish women's soccer players. Int J Environ Res Public Health.

[36] Arnason A, Gudmundsson A, Dahl HA, Jóhannsson E (1996). Soccer injuries in Iceland. Scand J Med Sci Sports.

[37] Bjørneboe J, Flørenes TW, Bahr R, Andersen TE (2011). Injury surveillance in male professional football; is medical staff reporting complete and accurate. Scand J Med Sci Sports.

[38] Brynhildsen J, Lennartsson H, Klemetz M, Dahlquist P, Hedin B, Hammar M (1997). Oral contraceptive use among female elite athletes and age-matched controls and its relation to low back pain. Acta Obstet Gynecol Scand.

[39] Brynhildsen J, Hammar J, Hammar ML (1997). Does the menstrual cycle and use of oral contraceptives influence the risk of low back pain A prospective study among female soccer players. Scand J Med Sci Sports.

[40] Cabral LMC (2017). Lesões músculo-esqueléticas em atletas de alta competição.

[41] Çali A, Gelecek N, Subasi SS (2013). Non-specific low back pain in male professional football players in the Turkish super league. Sci Sports.

[42] Cesca D, Daronco LSE, Sá A, Denardini V, Borges L, Balsan LAG (2012). Histórico de lesões, avaliação postural e dor musculoesquelética em atletas de futebol. Rev Salusvita.

[43] Coelho MM (2011). Prevalência de lesões em atletas de futebol profissional de duas equipes catarinense.

[44] Dupont G, Nedelec M, McCall A, McCormack D, Berthoin S, Wisløff U (2010). Effect of 2 soccer matches in a week on physical performance and injury rate. Am J Sports Med.

[45] Eirale C, Hamilton B, Bisciotti G, Grantham J, Chalabi H (2012). Injury epidemiology in a national football team of the Middle East. Scand J Med Sci Sports.

[46] Ekstrand J, Hägglund M, Waldén M (2011). Epidemiology of muscle injuries in professional football (soccer). Am J Sports Med.

[47] Ekstrand J, Hägglund M, Fuller CW (2011). Comparison of injuries sustained on artificial turf and grass by male and female elite football players. Scand J Med Sci Sports.

[48] Ekstrand J, Krutsch W, Spreco A, van Zoest W, Roberts C, Meyer T, Bengtsson H (2020). Time before return to play for the most common injuries in professional football a 16-year follow-up of the UEFA Elite Club Injury Study. Br J Sports Med.

[49] Escobar CLS (2018). Lesiones deportivas en futbolistas durante el torneo clausura 2017: Club Deportivo Petapa FC, Guatemala, Septiembre 2018.

[50] Grosdent S, Demoulin C, Rodriguez de La Cruz C.Giop R.Tomasella M.Crielaard JM.Vanderthommen M (2016). Lumbopelvic motor control and low back pain in elite soccer players a cross-sectional study. J Sports Sci.

[51] Hägglund M, Waldén M, Ekstrand J (2009). Injuries among male and female elite football players. Scand J Med Sci Sports.

[52] Hägglund M (2007). Epidemiology and prevention of football injuries.

[53] Hides JA, Oostenbroek T, Smith MMF, Mendis MD (2016). The effect of low back pain on trunk muscle size/function and hip strength in elite football (soccer) players. J Sports Sci.

[54] Junge A, Dvorak J, Chomiak J, Peterson L, Graf-Baumann T (2000). Medical history and physical findings in football players of different ages and skill levels. Am J Sports Med.

[55] Kristenson K, Bjørneboe J, Waldén M, Andersen TE, Ekstrand J, Hägglund M (2013). The Nordic Football Injury Audit higher injury rates for professional football clubs with third-generation artificial turf at their home venue. Br J Sports Med.

[56] Krutsch W, Memmel C, Alt V, Krutsch V, Tröß T.Aus der Fünten K.Meyer T (2022). Timing return-to-competition a prospective registration of 135 different types of severe injuries in Germany's highest football league. Arch Orthop Trauma Surg.

[57] Larruskain J, Lekue JA, Diaz N, Odriozola A, Gil SM (2018). A comparison of injuries in elite male and female football players a five-season prospective study. Scand J Med Sci Sports.

[58] Lu D, McCall A, Jones M, Kovalchik S, Steinweg J, Gelis L, Duffield R (2020). Injury epidemiology in Australian male professional soccer. J Sci Med Sport.

[59] Nascimento GARL, Borges MGL, Souza PVN, Sanches DL, Furtado JM (2015). Lesões musculoesqueléticas em jogadores de futebol durante o Campeonato Paraense de 2013. Revista Brasileira de Futsal e Futebol.

[60] C D, Arliani GG, Thiele ES, Cat MNL, Cohen M, Pagura JR (2019). Prospective evaluation of injuries occurred during the Brazilian Soccer Championship in 2016. Rev Bras Ortop.

[61] C D (2017). Lesões em jogadores de futebol durante os jogos do campeonato brasileiro da série A.

[62] Noormohammadpour P, Aghaei-Afshar M, Mansournia MA, Mirzashahi B, Akbari-Fakhrabadi M, Linek P (2020). The relationship between low back pain incidence and ultrasound assessment of trunk muscles in adult soccer players a cohort study. Asian J Sports Med.

[63] Pangrazio O, Forriol F (2016). Diferencias de las lesiones sufridas en 4 campeonatos sudamericanos de fútbol femenino y masculino. Revista Latinoamericana de Cirurgía Ortopédica.

[64] Papacostas M, Pafis G, Bikos C, Porfiriadou A (2009). Athletic injuries in soccer: three year study of a Greek professional team. Physical Training.

[65] Paus V, Compadre P, Torrengo F (2003). Incidencia de lesiones en jugadores de fútbol profesional. Rev Asoc Argent Traumatol Deporte.

[66] Pedrinelli A, Cunha GAR, Thiele ES, Kullak OP (2013). Epidemiological study on professional football injuries during the 2011 Copa America, Argentina. Rev Bras Ortop.

[67] Peterson L, Junge A, Chomiak J, Graf-Baumann T, Dvorak J (2000). Incidence of football injuries and complaints in different age groups and skill-level groups. Am J Sports Med.

[68] Poulsen TD, Freund KG, Madsen F, Sandvej K (1991). Injuries in high-skilled and low-skilled soccer a prospective study. Br J Sports Med.

[69] Santos RMB, Gouveia FMV, Lima JE, Azevedo AF (2009). Análise epidemiológica das lesões em atletas de futebol profissional do Sport Club do Recife em 2007. EFDeportes.

[70] Silva AA, Dória DD, Morais GA, Prota RVM, Mendes VB, Lacerda AC (2005). Fisioterapia esportiva: prevenção e reabilitação de lesões esportivas em atletas do América Futebol Clube.

[71] Stubbe JH, van Beijsterveldt AMMC, van der Knaap S, Stege J, Verhagen EA, van Mechelen W, Backx FJG (2015). Injuries in professional male soccer players in the Netherlands a prospective cohort study. J Athl Train.

[72] Todeschini K, Daruge P, Bordalo-Rodrigues M, Pedrinelli A, Busetto AM (2019). Imaging assessment of the pubis in soccer players. Rev Bras Ortop.

[73] Torrontegui-Duarte M, Gijon-Nogueron G, Perez-Frias JC, Morales-Asencio JM, Luque-Suarez A (2020). Incidence of injuries among professional football players in Spain during three consecutive seasons a longitudinal, retrospective study. Phys Ther Sport.

[74] Tunås P, Nilstad A, Myklebust G (2015). Low back pain in female elite football and handball players compared with an active control group. Knee Surg Sports Traumatol Arthrosc.

[75] van Beijsterveldt AM, Stubbe JH, Schmikli SL, van de Port IG, Backx FJ (2015). Differences in injury risk and characteristics between Dutch amateur and professional soccer players. J Sci Med Sport.

[76] Vasconcelos J, Assis TO (2010). Lesões em atletas de futebol profissional de um clube da cidade de Campina Grande, no Estado da Paraíba. Rev Bras Cienc Saude.

[77] Waldén M, Hägglund M, Ekstrand J (2005). UEFA Champions League study a prospective study of injuries in professional football during the 2001-2002 season. Br J Sports Med.

[78] Waldén M, Hägglund M, Ekstrand J (2007). Football injuries during European Championships 2004-2005. Knee Surg Sports Traumatol Arthrosc.

[79] Waldén M, Hägglund M, Orchard J, Kristenson K, Ekstrand J (2013). Regional differences in injury incidence in European professional football. Scand J Med Sci Sports.

[80] Wilson F, Ardern CL, Hartvigsen J, Dane K, Trompeter K, Trease L (2021). Prevalence and risk factors for back pain in sports a systematic review with meta-analysis. Br J Sports Med.

[81] Volpi P, Taioli E (2012). The health profile of professional soccer players future opportunities for injury prevention. J Strength Cond Res.

[82] Onaka GM, Gaspar-Jr JJ, Graças D, Barbosa FSS, Martinez PF, Oliveira-Junior SA (2017). Sports injuries in soccer according to tactical position a retrospective survey. Fisioter Mov.

[83] Rossi MK, Pasanen K, Heinonen A, Myklebust G, Kannus P, Kujala UM (2018). Incidence and risk factors for back pain in young floorball and basketball players a prospective study. Scand J Med Sci Sports.

[84] Newlands C, Reid D, Parmar P (2015). The prevalence, incidence and severity of low back pain among international-level rowers. Br J Sports Med.

[85] Gregory PL, Batt M, Kerslake RW (2004). Comparing spondylolysis in cricketers and soccer players. Br J Sports Med.

[86] Heneweer H, Vanhees L, Picavet HS (2009). Physical activity and low back pain a U-shaped relation?. Pain.

[87] Lewin G (1989). The incidence of injuries in an English professional soccer club during one competitive season. Physiotherapy.

[88] Kuorinka I, Jonsson B, Kilbom A, Vinterberg H, Biering-Sørensen F, Andersson G (1987). Standardized Nordic questionnaire for the analysis of musculoskeletal symptoms. Appl Ergon.

[89] Hides JA, Stanton WR, Mendis MD, Gildea J, Sexton MJ (2012). Effect of motor control training on muscle size and football games missed from injury. Med Sci Sports Exerc.

[90] Noormohammadpour P, Rostami M, Mansournia MA, Farahbakhsh F, Shahi MHP, Kordi R (2016). Low back pain status of female university students in relation to different sport activities. Eur Spine J.

[91] Nicholas JA, Hershman E (1990). The lower extremity and spine.

[92] Dvorak J, Junge A (2000). Football injuries and physical symptoms a review of the literature. Am J Sports Med.

[93] Junge A, Dvorak J (2000). Influence of definition and data collection on the incidence of injuries in football. Am J Sports Med.

[94] Ekstrand J (1982). Soccer injuries and their prevention.

[95] Schmidt-Olsen S, Jørgensen U, Kaalund S, Sørensen J (1991). Injuries among young soccer players. Am J Sports Med.

